# The association of dermatologist demographic density with melanoma survival in New South Wales, Australia

**DOI:** 10.1111/ajd.14113

**Published:** 2023-06-24

**Authors:** Stephanie C. Blake, Pascale Guitera, Anne E. Cust, Claire Galea, Serigne N. Lo, Richard A. Scolyer, Bruce K. Armstrong, John F. Thompson, Scott W. Menzies, Christine Madronio, Rachael L. Morton, Graham J. Mann, Caroline G. Watts

**Affiliations:** ^1^ School of Medicine University of New South Wales Sydney New South Wales Australia; ^2^ Melanoma Institute Australia The University of Sydney Sydney New South Wales Australia; ^3^ Sydney Melanoma Diagnostic Centre Royal Prince Alfred Hospital Sydney New South Wales Australia; ^4^ The Daffodil Centre The University of Sydney, A Joint Venture with Cancer Council NSW Sydney New South Wales Australia; ^5^ Sydney School of Public Health, Faculty of Medicine and Health The University of Sydney Sydney New South Wales Australia; ^6^ Faculty of Medicine and Health, Sydney Medical School The University of Sydney Sydney New South Wales Australia; ^7^ Tissue Pathology and Diagnostic Oncology Royal Prince Alfred Hospital and NSW Health Pathology Sydney New South Wales Australia; ^8^ Charles Perkins Centre The University of Sydney Sydney New South Wales Australia; ^9^ School of Population and Global Health, Population and Public Health The University of Western Australia Perth Western Australia Australia; ^10^ Department of Melanoma and Surgical Oncology Royal Prince Alfred Hospital Sydney New South Wales Australia; ^11^ NHMRC Clinical Trials Centre The University of Sydney Sydney New South Wales Australia; ^12^ The John Curtin School of Medical Research Australian National University Canberra Australian Capital Territory Australia; ^13^ Surveillance and Evaluation Research Program, Kirby Institute University of New South Wales Sydney New South Wales Australia

**Keywords:** delivery of health care, dermatologists, melanoma, survival

Dear Editor,

Most Australian dermatologists (92%) work in major cities,^1^ limiting dermatology access to people residing in urban areas and placing a burden on rural patients to travel to major centres for such care. In the United States, higher melanoma mortality has been observed in areas with low dermatologist density.^2^, ^3^ Whether a similar association exists in other populations is currently unknown. We aimed to assess the association of dermatologist density with melanoma survival in the state of New South Wales (NSW), Australia.

The Melanoma Patterns of Care (MPOC) study is a population‐based study of doctors' reported management of adult patients with a histological diagnosis of primary invasive cutaneous melanoma and a much smaller subset with in situ melanomas identified in the same period and notified to the NSW Cancer Registry between 23 October 2006 and 22 October 2007. Additional study details have been published previously.^4^ A follow‐up study has been conducted in which the MPOC data set was linked to the NSW Cancer Registry, the NSW Registry for Births, Deaths and Marriages and the Cause of Death Unit Record Files from NSW and the Australian Capital Territory, to collect additional cancer and mortality data up to December 2018, achieving a >10‐year follow‐up of all cases.

Patient characteristics included age, sex and residential postcode. Recorded features of diagnosed melanomas included Breslow thickness, histological subtype, site and type of biopsy performed and clinician characteristics included age and postcode of treating doctor. Remoteness from medical services was assessed using the Australian Standard Geographical Classification Remoteness Area Score (ASGC‐RA)^5^ and assigned to patients and clinicians based on their workplace or residence postcode at time of diagnosis, using 2006 Census data. Socio‐economic status was measured using the Index of Relative Socio‐Economic Disadvantage (ISRD)^6^ and postcode. Dermatologist density was assessed using the Australian Health Practitioners Registration Association (AHPRA) National Health Workforce Dataset from 2013.^7^ As the APHRA dataset did not provide density data when there were less than three dermatologists in a region, we also used an opt‐in database of workplace postcodes managed by the Australasian College of Dermatologists to estimate dermatologist density. The density (per 100,000 population) was mapped to regions from the Australian Statistical Geography standard, Statistical Area Level 4 (ASGC‐SAL4) from 2011, and categorised.^5^

Patients who had multiple primary melanomas had their thickest melanoma included, and those with in situ melanoma or with metastatic melanoma when diagnosed were excluded. The association between melanoma survival and dermatologist density was assessed in a multivariable analysis, adjusting for age, sex, patient's ASGC‐RA and IRSD, histological subtype and Breslow thickness. Analysis was conducted using SAS (version 9.3, SAS Institute Inc.), with statistical significance inferred at *p* < 0.05.

The analysis data set comprised 3160 patients (82% of the original MPOC study participants). Patient and tumour characteristics are summarised in Table 1, and their clinician characteristics are in Table 2. Of the participants, 7% died from melanoma and a further 27% died from other causes. 25% of melanomas were diagnosed by dermatologists, 36% by generalist general practitioners (GPs) and 16% by skin‐cancer specialised GPs (Table 2). Median follow‐up was 11.5 years. No differences in melanoma‐specific survival were observed between metropolitan, regional and remote populations. No significant association with melanoma‐specific survival was seen with increasing dermatologist demographic density (Table 3).

**TABLE 1:** Patient and melanoma characteristics.

	*N*	%
*Age (years)*		
<40	290	9
40–49	347	11
50–59	581	18
60–69	703	22
70–79	707	22
>80	532	17
*Sex*		
Male	1963	62
Female	1197	38
*Rurality score*		
Major cities	1326	42
Inner regional	1284	41
Outer regional	528	17
Remote	22	1
*Melanoma histological subtype*		
Superficial spreading	1638	52
Nodular	410	13
Lentigo maligna melanoma	319	10
Other	213	7
Not specified	580	18
Mean Breslow thickness (mm)	1.3 (IQR 0.4–1.4)	
*Socio‐economic status* ^a^		
1st Quintile 1 (lowest)	361	11
2nd Quintile	621	20
3rd Quintile	830	26
4th Quintile	522	17
5th Quintile (highest)	823	26
Missing	3	
Median follow‐up (years)	11.8 (IQR 11.5–12.1)	
*Survival status*		
Survivors	2094	66
Death from melanoma	217	7
Death from other causes	849	27

^a^ Socio‐economic status measured as the Index of Relative Socio‐economic Disadvantage.

**TABLE 2:** Primary clinician demographics and dermatologist density per 100,000 population in NSW.

	*N*	%
*Diagnosing clinician*		
Generalist general practitioner (GP)	833	36
Dermatologist	575	25
GP in skin cancer clinic	367	16
General surgeon	322	14
Plastic surgeon	144	6
Other	20	1
Not provided	899	
*Clinician location*		
Major city	1181	52
Inner regional	769	34
Outer regional	305	13
Remote	6	0
Not provided	899	
*Dermatologist density per 100,000 population*		
0.0	270	9
0.01–0.99	879	28
1.0–2.0	688	22
2.1–3.0	889	28
>4.0	427	14
Missing	7	

**TABLE 3:** Melanoma survival associated with dermatologist density and adjustment factors: crude and adjusted hazard ratios and 95% confidence intervals.

Variable	Crude hazard ratio	Adjusted hazard ratio^a^		
	HR (95% CI)	*p*‐Value	HR (95% CI)	*p*‐Value
*Dermatologist density per 100,000 population*				
0.0	1	0.95	1	0.63
0.01–0.99	1.08 (0.63, 1.85)		0.94 (0.53, 1.65)	
1.0–2.0	1.19 (0.69, 2.07)		1.19 (0.65, 2.18)	
2.1–3.9	1.05 (0.61, 1.80)		1.13 (0.63, 2.01)	
≥4.0	1.14 (0.63, 2.06)		1.55 (0.77, 3.10)	
*Melanoma subtype*				
Superficial spreading	1	<0.001	1	0.21
Nodular	5.24 (3.80, 7.21)		1.51 (1.03, 2.22)	
Lentigo maligna	1.00 (0.55, 1.80)		1.21 (0.66, 2.19)	
Not specified	1.22 (0.80, 1.86)		1.10 (0.71, 1.69)	
Other	2.70 (1.69, 4.32)		1.00 (0.60, 1.67)	
*Breslow thickness*				
0.1–0.7 mm	1	<0.001	1	<0.001
0.8–1.0 mm	2.36 (1.42, 3.90)		2.31 (1.39, 3.84)	
1.1–2.0 mm	3.99 (2.62, 6.08)		3.73 (2.41, 5.77)	
2.1–4.0 mm	8.90 (5.96, 13.31)		7.58 (4.83, 11.91)	
>4.0 mm	19.35 (12.73, 29.40)		16.24 (9.92, 26.59)	
*Patient remoteness*				
Major city	1	0.51	1	0.32
Inner regional	0.85 (0.63, 1.15)		0.93 (0.65, 1.33)	
Outer regional	1.11 (0.77, 1.60)		1.40 (0.84, 2.33)	
Remote	1.30 (0.32, 5.29)		0.97 (0.22, 4.20)	
*Socio‐economic status* ^b^ *‐patient*				
1st Quintile (lowest)	1	0.57	1	0.52
2nd Quintile	1.06 (0.66, 1.71)		1.17 (0.72, 1.91)	
3rd Quintile	0.91 (0.57, 1.45)		0.90 (0.56, 1.45)	
4th Quintile	0.89 (0.54, 1.48)		0.97 (0.56, 1.67)	
5th Quintile (highest)	0.77 (0.48, 1.23)		0.78 (0.45, 1.33)	

^a^ Multivariable model adjusted for age, sex, patient's Australian Statistical Geographical Classification—Remoteness Area (ASGC‐RA), and Index of Relative Socio‐economic Disadvantage (IRSD), histological type and Breslow thickness. ASGC‐RA scores were calculated using ARIA (Accessibility/Remoteness index of Australia) scores, which are based on postcode and measure access to goods, services and opportunity for social interactions and potential limitations on these.^5^

^b^ Socio‐economic status measured as the Index of Relative Socio‐economic Disadvantage.

In a US study, Aneja et al.^2^ found that the presence of 0.001 to 1 dermatologist/100,000 population was associated with a 35% reduction in melanoma‐specific mortality (95% CI 13%–57%) compared with no dermatologists. Another study found that an increase in dermatologists increased the odds of earlier diagnosis: OR 1.4 for early‐stage (in situ, locally confined) vs late‐stage (regional or distant metastasis) diagnosis per additional dermatologist/10,000 population, (95% CI 1.1–1.7, *p* = 0.01).^3^ In our study, while GPs performed the majority of initial excisions for melanoma, only 27% of GPs who did the excision biopsy also did the definitive wide local excision.^9^ Limitations of the study include that we may not have captured all dermatologists working in NSW, but believe we had good coverage, as the database used is managed by the only accredited college for the training and continuing professional development of dermatologists in Australia. Secondly, advances have been made in melanoma care since the study period, with increasing use of immunotherapies improving outcomes for patients with later‐stage disease.

There is considerable variation in Australian GPs' confidence and engagement in managing melanoma,^10^ GP‐led melanoma care in Australia has been reported to be aligned with current guidelines and yields similar survival rates to the national average.^8^ Whereas in the US family physicians do not routinely perform skin exams or biopsies,^11^ Australian GPs and dermatologists are each well placed to offer skin checks, which are associated with diagnosis of thinner melanomas and lower mortality.^12^

These reasons, and the universal health care available in Australia, may help explain the lack of association we observed between dermatologist demographic density and melanoma survival.

## FUNDING INFORMATION

This work was supported by the National Health and Medical Research Council (NHMRC) Centre of Research Excellence in Melanoma Grant #1135285 and Project Grant #1165936; Cancer Institute NSW (05/POC/1‐06), and the NSW State Government via a grant to the NSW Melanoma Network. Additional financial and in‐kind support was provided by Melanoma Institute Australia and the NSW Melanoma Network. RAS is supported by a NHMRC Investigator Grant (#2018514). RLM is supported by an NHMRC Investigator Grant (#1194703) and a University of Sydney Robinson Fellowship. RAS, GJM and JFT were recipients of an NHMRC Program Grant (1093017). AEC is supported by a NHMRC Investigator Grant (#2008454).

## CONFLICT OF INTEREST STATEMENT

RAS has received fees for professional services from MetaOptima Technology Inc., F. Hoffmann‐La Roche Ltd, Evaxion, Provectus Biopharmaceuticals Australia, Qbiotics, Novartis, Merck Sharp & Dohme, NeraCare, AMGEN Inc., Bristol‐Myers Squibb, Myriad Genetics and GlaxoSmithKline. JFT has received honoraria for advisory board participation from BMS Australia, MSD Australia, GSK and Provectus Inc and travel and conference support from GSK, Provectus Inc and Novartis. Other authors have nothing to declare.

## ETHICAL APPROVAL

The Human Research Ethics Committees of the University of Sydney and Cancer Institute NSW approved this project.
